# Host Susceptibility Factors to Bacterial Infections in Type 2 Diabetes

**DOI:** 10.1371/journal.ppat.1003794

**Published:** 2013-12-26

**Authors:** Yunn-Hwen Gan

**Affiliations:** Department of Biochemistry, Yong Loo Lin School of Medicine, Immunology Program, National University of Singapore, Singapore; University of North Carolina at Chapel Hill School of Medicine, United States of America

## Introduction

Globally, the number of people with Type 2 diabetes (T2D) or diabetes mellitus is projected to grow to 366–440 million by 2030, with three quarters of the increase in low- to middle-income countries [1]. The burden of communicable diseases is concentrated in low-income and resource-strapped regions, and one could predict that diabetes-related infections will rise significantly in these areas. Given the complex etiology of T2D and its many associated complications, the mechanisms underlying the association between diabetes and bacterial infections are poorly understood.

## What Bacterial Infections Are Highly Associated with Type 2 Diabetes?

Diabetic foot ulcers are a common complication of diabetes frequently associated with the presence of *Staphylococcus aureus* [2], as are chronic leg ulcers, surgical site infections, and chronic wounds [3]–[4]. Furthermore, invasive staphylococcal infections such as endocarditis or bacteremia are more prevalent in diabetic than in non-diabetic patients and are associated with a poorer outcome in diabetic patients [5]–[6]. Invasive group B streptococcal infections are also more common in diabetics [7]. In East Asia, diabetes is a well-known risk factor for *Klebsiella pneumoniae* liver abscess, and those with uncontrolled glycemia (HbA1c≥7%) are at higher risk of metastatic complications [8]. Furthermore, there is increased prevalence of urinary tract infection (UTI) and 1.4 times higher recurrence rate in women with T2D [9].

The association between tuberculosis (TB) and T2D is still debated. The relative risk estimates of diabetics associated with TB vary considerably from 1.2 to 7.8, with the lowest estimates reported in larger studies and in low–TB-burden countries [1]. Some studies have shown that poor glycemic control is associated with increased TB risk [10]–[11] but not others [12]. It could be that the association is evident only in high–TB-burden areas and populations with significant latent TB cases.

In contrast to TB, up to 60% of melioidosis patients have underlying T2D [13]. This makes melioidosis the most highly associated bacterial infection with diabetes. Melioidosis is endemic in Southeast Asia and Northern Australia and caused by the gram-negative bacterium *Burkholderia pseudomallei*.

## How Does Diabetes Affect the Response of Innate Immune Cells Such as Phagocytes to Infection?

A defective innate immune response is assumed to underlie diabetics' susceptibility to bacterial infections. It is generally accepted that phagocytic functions such as chemotaxis, phagocytosis, and respiratory burst are impaired, although evidence is conflicting, likely due to the use of different models and methodology. The responses seen in various diabetic mouse models sometimes add to the confusion because they do not accurately approximate diabetes in humans. To answer this question directly, I believe we should examine the evidence documenting the interaction between the human diabetic innate immune response with specific pathogens known to be associated with T2D. In melioidosis, polymorphonuclear cells (PMNs) from diabetic subjects exhibited decreased phagocytosis and chemotaxis in response to *Burkholderia pseudomallei* [14] and were defective in the release of extracellular DNA, also known as neutrophil extracellular traps (NETS), resulting in poorer bacterial killing [15]. In TB, where alveolar macrophages and monocytes are recognized as important effector cells for containment, diabetic monocytes showed impaired chemotaxis in response to the bacteria, and alveolar macrophages in TB patients with diabetes were less activated, with decreased hydrogen peroxide production [reviewed in 1]. However, these studies did not address the mechanisms underlying these immune defects. A recent study found reduced association of *Mycobacterium tuberculosis* with monocytes from diabetic patients that involved serum proteins [16]. The authors speculated that reduced opsonisation and phagocytosis of the bacterium could be due to defective complement proteins or complement receptors found in the diabetics.

## How Does Glycemic Control Influence Susceptibility to Infection?

Evidence from mice and humans shows that diabetics with carefully controlled blood glucose were no more susceptible to bacterial infections than healthy controls [17]–[20]. Acute hyperglycemia in humans has been shown to reduce neutrophil degranulation in response to lipopolysaccharides (LPS) [21]. An important consequence of poor glucose control is the formation of excessive glycation end products, which is strongly dependent on the concentration and reactivity of the reducing sugars. Glycation occurs non-enzymatically between free carbonyls of sugars and amino groups of macromolecules by the Maillard reaction [22], and highly glycated proteins will be present abundantly in diabetics with poor glycemic control. In vitro studies show that high glucose concentrations, typical of those found in diabetes, inhibited C-type lectin binding to high mannose glycoprotein and binding of DC-SIGN to fucosylated ligand. Complement activation via the mannan binding lectin pathway was also inhibited by high glucose concentrations [23]. The authors proposed that high glucose in diabetics could disrupt C-type lectin function via competitive inhibition of carbohydrate binding, contributing to poor innate immune response to infection. In another study showing that glycated bovine serum albumin resulted in a loss of affinity for siderophore scavengers, the authors proposed that glycated human serum albumin and other serum proteins such as lipocalin-2 and transferrin may disrupt the innate immune response and promote bacterial infection in diabetics by increasing the bioavailability of limiting nutrients such as iron, thus enhancing bacterial growth [24].

A small proportion of glycated products undergo further slow and irreversible chemical rearrangements, including oxidation, to form advanced glycation end products (AGEs) [22]. When AGE albumin was used as an example of the effect of AGEs on human neutrophils, the authors found that AGE albumin bound to the receptor for AGE (RAGE) present on neutrophils. This binding inhibited transendothelial migration and *S. aureus*-induced production of reactive oxygen species, resulting in impaired bacterial killing [25].

In UTI, urosepsis patients had higher levels of glycated hemoglobin (HbA1c), and this is an independent determinant for occurrence of urosepsis [26]. It has been shown that the increased prevalence of UTI in diabetic women is not the result of differences in virulence of the *Escherichia coli* strains but due to an increased adherence of Type 1 piliated *E. coli* [27]. Not only was the adherence of Type 1 piliated *E. coli* higher in diabetic than in control epithelial cells, the degree of bacterial adherence correlated with the HbA1c levels in the blood of diabetic patients [28]. FimH is the Type 1 fimbrial tip adhesin and invasin of *E. coli*. Taganna et al. proposed that changes in the N-glycosylation profiles in the uroepithelial cell receptors for FimH to more of the high-mannose type glycans, to which FimH has an unusually high affinity [27], may increase susceptibility [29].

In respiratory infections with *S. aureus*, elevation of basolateral glucose concentration in human airway epithelial cells and blood glucose in diabetic mice promoted airway *S. aureus* growth, whereas pretreatment with the anti-diabetic drug metformin inhibited the growth [30]. It is tempting to extrapolate this finding to explain why hyperglycemia may promote airway infection with *S. aureus*.

Therefore, hyperglycemia results in protein glycation and formation of AGEs, which can have a diverse impact on host cell function. It can cause impairment of host proteins involved in complement activation, bacterial uptake, phagocytic killing, and scavenging of biolimiting nutrients and change the binding of host surface receptors for pathogens.

## How Does Oxidative Stress Influence Susceptibility to Infection?

**Figure 1:**
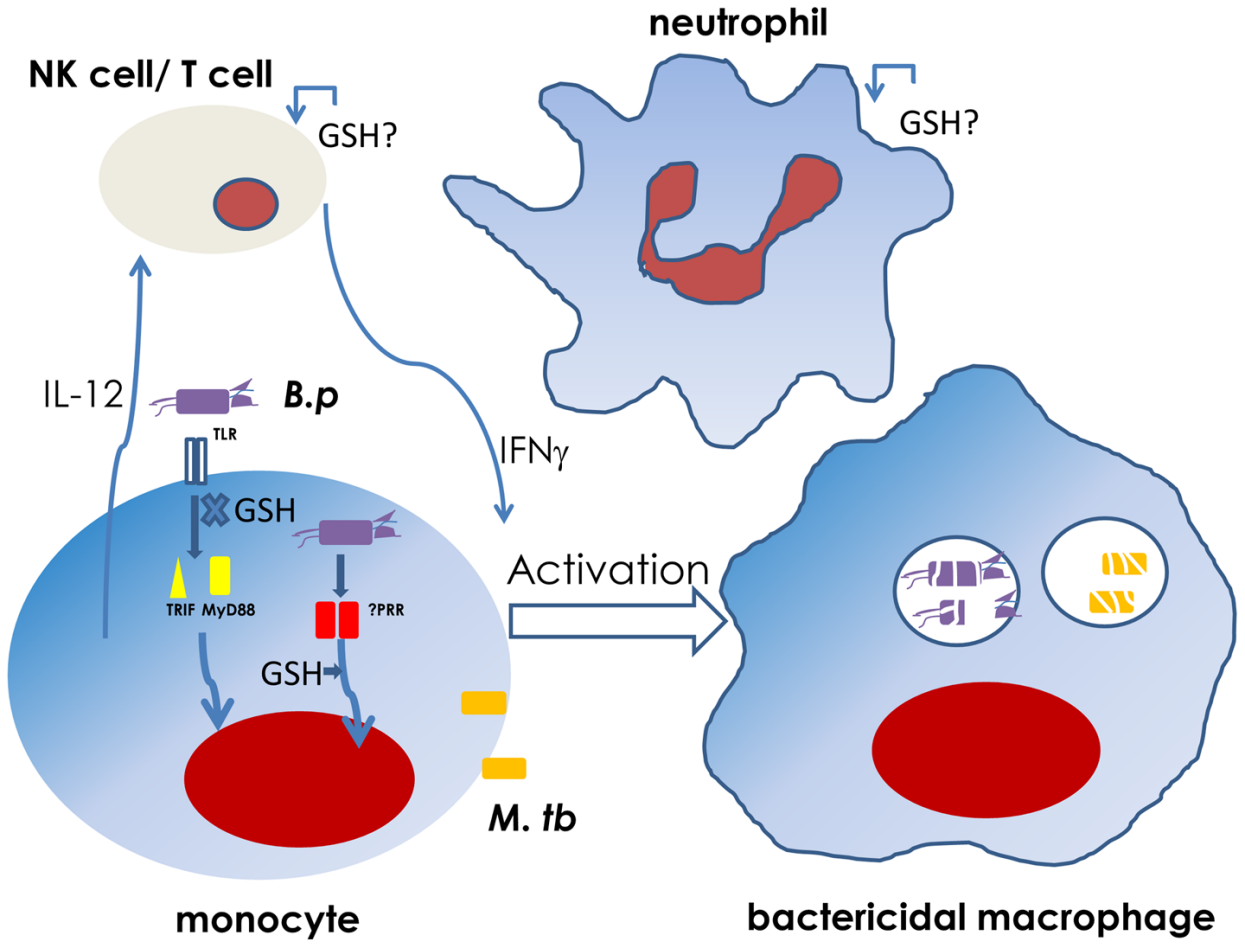
Glutathione ratio affects intracellular bacterial control via the IL-12–IFNγ axis [20]. A decreased glutathione ratio in monocytes results in reduced IL-12 production. IL-12 induces the production of IFNγ from natural killer (NK) and T cells. IFNγ is necessary to activate the infected monocytes to become bactericidal for killing of intracellular *B. pseudomallei* (*B. p.*) and *M. tuberculosis (M. tb.)*. The IL-12 response from monocytes infected with *B. p.* is dependent on TRIF and MyD88 adaptors, yet these signaling pathways do not seem to be affected by the GSH ratio [20]. Perhaps another signaling pathway mediated by an intracellular pattern recognition receptor (PRR) is also necessary for IL-12 induction, and this pathway is modulated by GSH. The effect of GSH on diabetic NK cell and neutrophil function in response to *B. p.* is currently under investigation. In tuberculosis, indirect evidence where GSH was added to healthy NK cells and neutrophils in vitro shows increased cellular control of *M. tb.* [33].

Glutathione exists in reduced or oxidized form and is the major redox regulator in our cells and tissues. A decreased ratio of reduced (GSH) to oxidized (GSSG) glutathione is indicative of oxidative stress. T2D patients with poor glycemic control have a decreased GSH ratio [20]. This can be contributed to by various mechanisms, such as the induction of superoxide and hydrogen peroxide by AGEs, glucose affecting GSH synthesis, and by excess glucose being converted to sorbitol via the polyol pathway, thereby consuming NADPH in the process. As NADPH is a cofactor required for GSH regeneration from GSSG, the consumption of NADPH affects the regeneration of GSH [31]. We found that peripheral blood mononuclear cells (PBMCs) and monocytes from T2D patients with poor glycemic control (HbA1c≥8) were defective in IL-12 production in response to *B. pseudomallei* and *M. tuberculosis*. Defective IL-12, in turn, impaired IFNγ production and the bactericidal activity of monocytes against these intracellular bacteria [20]. Defective IL-12 production in diabetics was only against these two bacteria but not *Salmonella* or Toll-like receptor (TLR) ligands and could be reversed by improving the glutathione ratio. It is interesting that whole blood gene expression profiles of both melioidosis and tuberculosis patients were indistinguishable, defined by an 86-gene signature dominated by the Type 1 and 2 IFN pathways [32], implying shared signaling pathways induced by these two bacteria. We do not yet know how GSH influences IL-12 production in these infections, but GSH could modulate intracellular free radicals, maintain the thiol status of proteins, and covalently bind cysteine moieties in cytosolic proteins (S-glutathionylation), thereby potentially affecting the function of signaling intermediates, transcription factors, or cofactors. Thus, oxidative stress due to poor glucose control results in a decreased GSH ratio, impairing the IL-12 and IFNγ responses, which are critical for effective bacterial control in these intracellular bacterial infections (Figure 1). As IL-12 is important for the priming and differentiation of T helper 1 (Th1) cells involved in protective cell-mediated immunity against these bacteria, it is highly possible that the adaptive immune response to these bacteria will be impaired in diabetics. This is supported indirectly through evidence where increasing the intracellular GSH favored a Th1 response, resulting in better *M. tuberculosis* control [reviewed in 33].

## Perspective

Host susceptibility factors contributing to increased risk of bacterial infections in T2D will undoubtedly be multifactorial, depending on tissue tropism, the type of protective immune response required and the unique life cycles of various pathogens. We are only beginning to understand the molecular mechanisms underlying susceptibility, and future research will reveal how the diabetic state, involving hyperglycemia and chronic oxidative stress, modifies tissues and organs such as the bladder, airway, liver, skin, and blood cells in different ways that encourage bacterial pathogenesis.
